# Circ_0011129 Encapsulated by the Small Extracellular Vesicles Derived from Human Stem Cells Ameliorate Skin Photoaging

**DOI:** 10.3390/ijms232315390

**Published:** 2022-12-06

**Authors:** Yu Zhang, Manqi Zhang, Amin Yao, Yalin Xie, Jingxiong Lin, Farooqi Sharifullah, Yixin Hong, Hongbo Chen, Fang Cheng, Wei Lai

**Affiliations:** 1 Department of Dermato-Venereology, The Third Affiliated Hospital of Sun Yat-sen University, Guangzhou 510000, China; 2 School of Pharmaceutical Sciences (Shenzhen), Shenzhen Campus of Sun Yat-sen University, Shenzhen 518107, China; 3 Department of Plastic Surgery, Sun Yat-sen University, Guangzhou 510000, China

**Keywords:** skin photoaging, small extracellular vesicles, circle RNA, human adipose-derived stem cells

## Abstract

Photoaging is not only the main cause of skin aging caused by exogenous factors, it is also related to a variety of skin diseases and even malignant tumors. Excessive and repeated exposure to ultraviolet radiation, especially UVA induces oxidative stress, DNA damage, inflammation, and collagen and elastin degeneration, ultimately leads to skin photoaging, manifested by skin redness, coarse wrinkles, and pigmentation even skin cancer. There has been a large demand of effective prevention and medications but approaches in the current management of photoaging are very limited. In the previous study, we found that a non-coding circular RNA circ_0011129 acts as a miR-6732-5p adsorption sponge to inhibit the reduction of type I collagen and the denaturation and accumulation of elastin in UVA-induced HDF cells photoaging model. However, in vivo instability and efficient delivery to the target cell of circRNA is a major challenge for its clinical application. Therefore, improving its stability and delivery efficiency are desired. In this study, we proposed a strategy of delivering circ_0011129 with small extracellular vesicles (sEVs) from human adipose-derived stem cells (hADSCs) to intervene in the photoaging process. The results showed that sEVs from hADSCs in 3D bioreactor culture (3D-sEVs) can prevent photoaging. Consequently, by overexpressing circ_0011129 in hADSCs, we successfully loaded it into 3D-sEVs (3D-circ-sEVs) and its protective effect was better. Our studies provide a novel approach to preventing skin photoaging, which has important clinical significance and application value for the development of non-coding RNA drugs to treat skin photoaging. We first screened out hADSCs-derived sEVs with excellent anti-oxidant effects. We then compared the sEVs collected from traditional 2D culture with 3D bioreactor culture. By miRNA-seq and GEO data analysis, we found that miRNAs in 3D-sEVs were enriched in cell activities related to apoptosis, cellular senescence, and inflammation. Subsequently, we prepared circ_0011129-loaded 3D-sEVs (3D-circ-sEVs) by overexpressing it in hADSCs for the treatment of photoaging in vitro. We proved that 3D-circ-sEVs can interfere with the process of cell photoaging and protect cells from UVA radiation damage, as well as in a H2O2-induced oxidative stress model.

## 1. Introduction

Up to 80% of accelerated skin aging process can be attributed to repeated excessive ultraviolet (UV) exposure, which has been called skin photoaging [1]. UV radiation induces increased production of reactive oxygen species (ROS) and inflammatory factors, ultimately leading to DNA damage and inflammation [2,3]. Moreover, there is a sustained up-regulation of matrix metalloproteinases (MMPs) in skin cells, especially MMP1, MMP3, and MMP9. MMPs promote degradation of collagen and elastin, facilitating decreased skin elasticity, skin roughening, and wrinkle formation processes [2,4]. Our group has been working primarily on skin photoaging with a focus on the Cathepsins family. It is generally believed internationally that MMPs are the main enzymes related to skin photoaging. However, some changes of skin photoaging, such as the degeneration and accumulation of elastin cannot be explained by the role of MMPs. Our group first reported the role of Cathepsins family in skin photoaging internationally, and confirmed that Cathepsin K is the most important enzyme to degrade elastin; additionally, we found that UVA is capable of increasing Cathepsin K expression in HDFs, most likely by activation of MAPK pathway and of AP-1, which has been shown to be the case for MMPs [5]. The increase of Cathepsin K level is an important reason for the decrease of elastin and the degeneration and accumulation of elastin. The histopathological changes of photoaging are mainly manifested in the decrease and disorder of collagen, as well as the degeneration and accumulation of elastin. Therefore, photoaged skin typically appears leathery and lax, with coarse wrinkles, telangiectasia, and pigmentation in clinical, seriously affects the patients’ appearance [6,7,8]. Skin photoaging is also associated with a variety of light-related skin diseases and skin malignant tumors, such as melanoma [9,10,11,12]. Thus, the intervention of skin photoaging has important clinical significance.

At present, there is a lack of safe, effective, and convenient anti-aging products for the prevention and treatment of skin photoaging. In recent years, the application of non- coding RNA in disease intervention and regulation of functional gene expression has become a research hotspot [13]. Non-coding RNA includes RNA with known functions, such as tRNA, lncRNA, microRNA, and circRNA, and RNA with unknown functions. Circular RNA (circRNA) is a ring-shaped closed structure formed by reverse splicing, without a 5′ end cap and a 3′ end poly-A tail, so it is not easy to be degraded by nuclease in vivo. circRNA can adsorb miRNA like a sponge and regulate the expression of miRNA target genes through base complementary pairing [13,14]. Nowadays, circRNA has been widely used in many fields. Studies have shown that circ-HSP90A promotes cell growth and immune escape in non-small cell lung cancer by regulating STAT3 signal transduction and programmed cell death 1 (PD-1)/PD-L1 checkpoint [15]. Accumulating evidence shows that circRNA is related to the origin and development of cutaneous malignant melanoma, and can be used as a biomarker for early diagnosis of melanoma [16]. We and other groups report that circRNA shows excellent application prospects in the intervention of skin photoaging, and we found for the first time that circCOL-ELNs can simultaneously regulate the synthesis and degradation of skin collagen and elastin, which have the conditions for clinical transformation [17,18]. However, instability in vivo and efficient delivery to the target cell of circRNA is a major challenge for its clinical application. Therefore, improving its stability and delivery efficiency is desired.

Interestingly, with the small size, high stability, lower cytotoxicity and immunogenicity, and target specificity, small extracellular vesicles (sEVs), also called exosomes, have a crucial role in distinct biological functions [19]. In recent years, due to the good histocompatibility and nontoxicity, sEVs secreted by mesenchymal stem cells (MSCs), such adipose tissue-derived MSCs and umbilical cord-derived MSCs are applied in the prevention and treatment of malignant tumors, cardiovascular and cerebrovascular diseases, diabetes, skin diseases, and other fields as ideal drug carriers [20,21,22]. Intriguingly, reports show that sEVs secreted by hADSCs have anti-aging effects, including anti-oxidant and anti-apoptosis, accelerating proliferation [23]. The latest research shows that three-dimensional (3D) cell culture can significantly improve the yield and functionality of sEVs, and sEVs secreted by HDF cells in 3D culture improved the skin thickness and collagen production in the nude mouse photoaging model [24]. The sEVs have good histocompatibility and non-toxic, so it is a safe and good biological carrier. At present, sEVs can selectively encapsulate targeted drugs, proteins, or nucleic acids. It can overcome the shortcomings of cytotoxicity, immunogenicity, low transfection rate, and so on when compared to lentivirus, liposomes, and other delivery vectors, and is considered to be the most promising drug and active ingredient delivery vector at present. Collectively, the combination therapy of hADSCs-sEVs carrying circRNA may be consider as a promising delivery of circRNA for treating skin photoaging.

To sum up, the best way to intervene skin photoaging is to regulate collagen and elastin at the same time. We have identified recently that circ_0011129 acts as a miR-6732-5p adsorption sponge to inhibit photoaging in vitro [25]. These suggested that circRNA has an excellent application prospect. However, instability in vivo and efficient delivery to the target cell of circRNA is a major challenge for its clinical application. Therefore, improving its stability and delivery efficiency are desired. The combination therapy of hADSCs-sEVs carrying circRNA may be consider as a promising delivery of circRNA for treating skin photoaging. Whether sEVs can be used as an efficient delivery vector of circ_0011129 and have anti-photoaging effect, which has not been reported in the literature. To explore the ability of sEVs can effectively carry circ_0011129 and explore their synergistic intervention in photoaging, we adopted 3D culture ADSCs-sEVs technology to improve the yield of exosomes, and established the sEVs vector of circ_0011129. The HDFs chronic photodamage model was used to study the effect of circ_0011129-loaded 3D-sEVs (3D-circ-sEVs) on chronic photodamage in HDFs, and lay a key technical foundation for the translation of the previous research results into clinical application.

## 2. Results

### 2.1. Cell-Conditioned Media from Human Adipose-Derived Stem Cells in 3D Bioreactor Culture Exibits the Best Anti-Photoaging Effect

**Figure 1:**
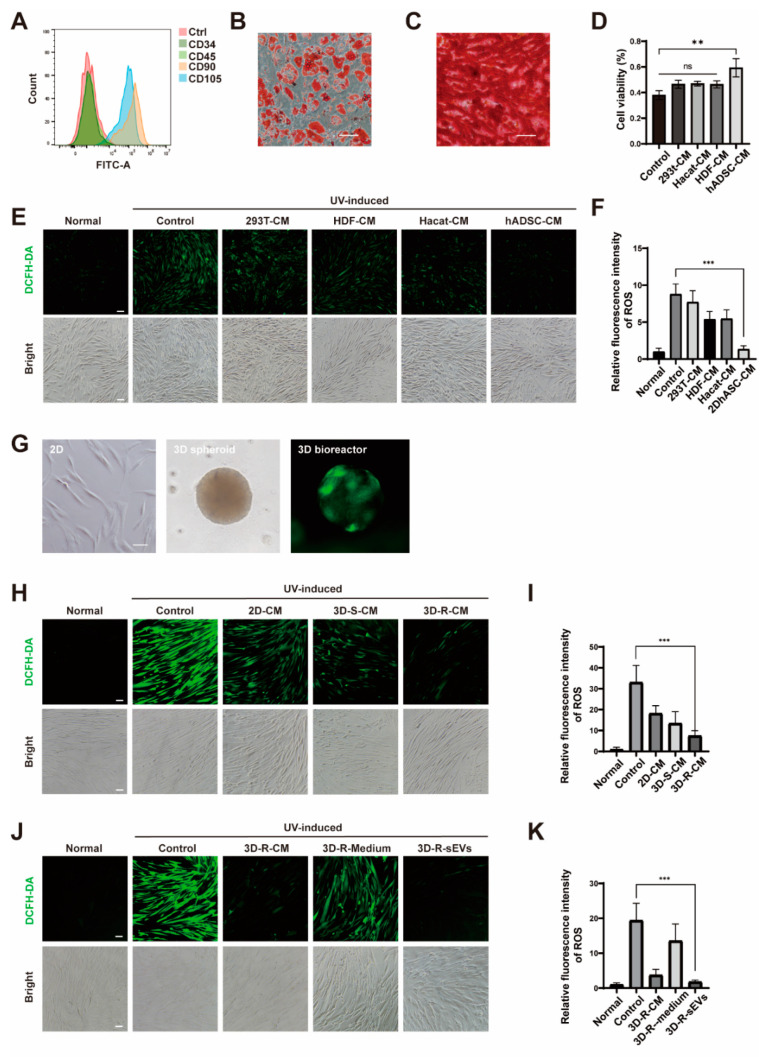
3D-R-sEVs has a significant anti-photoaging effect. (**A**) Flow cytometry analysis of CD34, CD45, CD90, and CD105 expression on hADSCs surface. (**B**) Oil Red O stain of adipo-differentiated hADSCs. Scale bar: 200 μΜ. (**C**) Alizarin Red stain of osteo-differentiated hADSCs. Scale bar: 200 μΜ. (**D**) showed the viability of HDF cells which pre-incubated with the conditioned medium (CM) of HEK-293T cells, HDF, HaCaT, and hADSCs, respectively, for 24 h after being exposed to 10 J/cm^2^ dose of UVA tested by CCK-8 (n = 3). (**E**,**H**,**J**) Representative images showed the level of ROS (green) in different treatment groups of HDF cells. Scale bar: 100 μΜ. (**F**,**I**,**K**) Relative quantitative analysis of fluorescence intensity of ROS in different treatment groups of HDF cells (n = 10). One-way ANOVA and Tukey post hoc test analyses were performed. Error bar, mean ± SD. ns represents not significant, ** *p* ≤ 0.01, *** *p* ≤ 0.001. (**G**) Morphology of hADSCs in 2D, 3D spheroid, and 3D bioreactor culture, respectively. Scale bar: 100 μΜ.

Studies have shown that conditioned medium (CM) from skin cells including HaCaT, HDF cells, and hADSCs have pro-proliferation and pro-migration functions [26]. As a hallmark of cellular senescence is stopping growth, we postulated that the CM of these cells may have anti-photoaging effect. To test this hypothesis, we isolated hADSCs from freshly discarded adipose tissue from healthy people and obtained hADSCs growing as spindle-shaped under the microscope. The purity of hADSCs was assessed by the mesenchymal stem cell markers CD90 and CD105 positively and CD34 and CD45 negatively (Figure 1A). After induction, hADSCs were successfully differentiated into the adipogenic lineage and osteogenic lineage (Figure 1B,C), indicating that hADSCs have the capability of pluripotent differentiation. Then, we collected the CM of hADSCs, HDF, and HaCaT, as well as HEK-293T cells, as a control. HDF as experimental cells were pre-incubated with these CMs for 24 h before exposure to a 10J/cm^2^ dose of UVA. It was found that hADSCs CM can enhance cell viability by CCK8 (Figure 1D). Next, HDF cells were preloaded with DCFH-DA, a ROS probe, to detect of ROS production during acquiring acute photoaging. The results of fluorescence microscopy showed that hADSCs CM induced the best resistance of HDF cells to acute photoaging (Figure 1E,F) and its protective effect on UVA-induced HDF cells was achieved by reducing the production of ROS. MSCs culture in 3D culture system can successfully mimic the microenvironment of a variety of tissue types. Among a variety of 3D culture systems, the spheroid model is a widely used multicellular 3D model due to the tendency of adherent cells to aggregate and allow heterogeneous cell populations to secrete more active components as compared to 2D cell culture. Compare to the simple spheroid model, 3D bioreactor culture is a physiologically relevant model that facilitate uniform cell distribution and increase mass transport by diffusion and convection using mixing systems of culture medium. Thus, we cultured hADSCs as spheroids in low adsorption plates and also attached to the microcarriers in a 3D bioreactor (Figure 1G). Then, we collected the CM of hADSCs produced under different culture methods, and compared their capacity to resisting to a dose of 15J/cm^2^ photoaging treatment. The results showed that the CM cultured in the 3D bioreactor (3D-R-CM) achieved the best anti-photoaging effect comparing with 2D and 3D spheroids culture (3D-S-CM) (Figure 1H,I). Previously, we and others have reported that MSCs secret small extracellular vesicles (MSC-sEVs) as cell-free vectors in alternative therapies for a variety of inflammatory diseases due to their potential to regulate inflammatory immune responses [27]. To test if the sEVs in the culture medium play a major role in anti-photoaging function, we isolated sEVs in 3D-R-CM by differential centrifugation, and found that sEVs is the major player of 3D-R-CM involved in anti-photoaging process (Figure 1J,K).

### 2.2. hADSCs-sEVs in 3D Bioreactor Culture Had an Optimal Anti-Photoaging Function In Vitro

**Figure 2:**
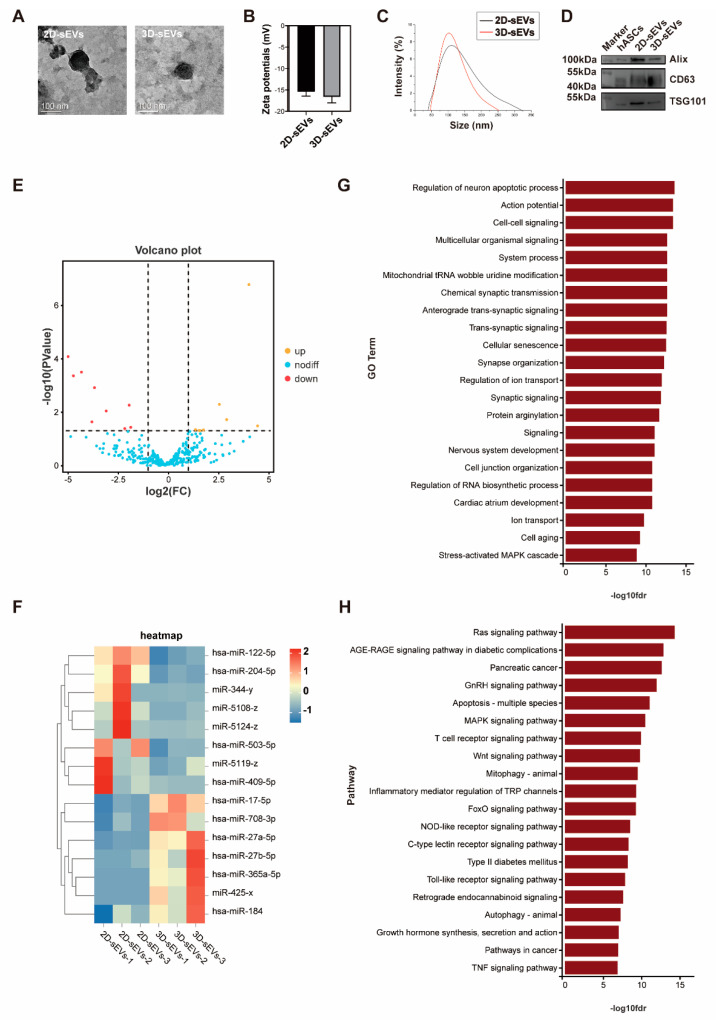
3D-sEVs contains anti-photoaging miRNAs. (**A**) Representative TEM images of 2D-sEVs and 3D-sEVs. Scale bar: 100 nm. (**B**,**C**) The size distribution and Zeta potential of 2D-sEVs and 3D-sEVs were measured by DLS (n = 3). (**D**) Western blotting of 2D-sEVs and 3D-sEVs for Alix, CD63, and TSG101. (**E**) The volcano plot representing significance of identified miRNAs which are differentially expressed with *p* < 0.05. The up-regulated miRNAs are shown in yellow, while down-regulated miRNAs are shown in red. (**F**) Heatmap of differentially expressed miRNAs in 2D-sEVs and 3D-sEVs. (**G**) Gene Ontology (GO) functional analysis of the differentially expressed miRNAs in 3D-sEVs compared with the 2D-sEVs. (**H**) KEGG enrichment analysis of the differentially expressed miRNAs in 3D-sEVs compared with the 2D-sEVs.

To explore the mechanisms of 3D-sEVs in alleviating cell aging, we first prepared hADSCs-sEVs from 2D and 3D-R-CM to compare the intrinsic biophysical properties between 2D-sEVs and 3D-sEVs. TEM imaging and DLS analysis showed spherical morphology of 2D-sEVs and 3D-sEVs (Figure 2A) with the average zeta potential of −20 mV (Figure 2B), and an average diameter was around 100 nm in both (Figure 2C), confirming that we successfully prepared hADSCs-sEVs in both 2D and 3D bioreactor culture. Furthermore, we identified the existence of extracellular vesicles-associated proteins Alix, CD63, and TSG101 (Figure 2D), which had no significant difference between 2D and 3D conditions. MicroRNAs (miRNAs) play important gene-regulatory roles in cell senescence mainly by blocking the translation of target mRNA, which may be involved in anti-photoaging function of hADSCs-sEVs. Therefore, we compared miRNA expression profiles in 3D-sEVs with those of 2D-sEVs by miRNA sequencing. The expression of eight miRNAs was significantly up-regulated and the expression of seven miRNAs was significantly down-regulated (fold changes ≥ 2, *p* < 0.05) (Figure 2E,F). We then performed gene enrichment analysis on the target genes of the above miRNAs differentially expressed in 2D-sEVs and 3D-sEVs through Miranda (v3.3a) and TargetScan (Version: 7.0) target gene databases. Gene Ontology (GO) survey showed that the differentially expressed genes were enriched in aging-related functions including apoptosis, cellular senescence, and inflammation (Figure 2G). KEGG enrichment analysis indicated the great potential of 3D-sEVs to regulate the MAPK, Ras and Wnt signal pathways (Figure 2H), implying that 3D bioreactor culture largely alters the miRNA expression profiles of hADSCs. These results indicated that compared with 2D-sEVs, 3D-sEVs exerted enhanced anti-photoaging efficacy in photoaged cell partially due to their differentially expressed miRNAs, and may serve as a potential therapeutic vector to improve current treatment for photoaging skin diseases.

### 2.3. Preparation and Characterization of sEVs from hADSCs Overexpressing circ_0011129 in 3D Bioreactor Culture

**Figure 3:**
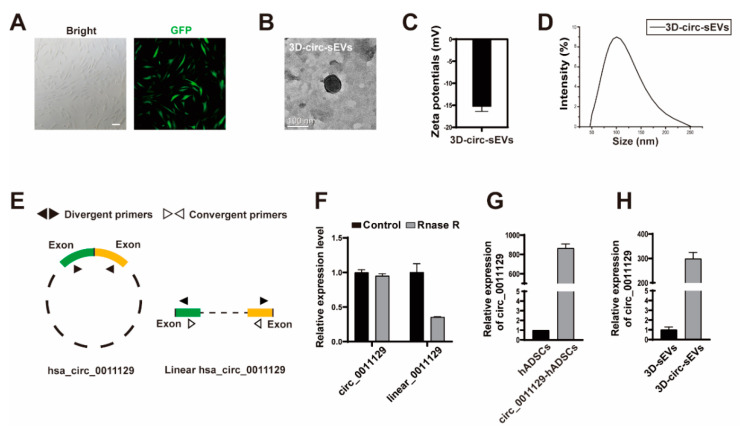
3D-sEVs can carry circ_0011129 expressed by hADSCs. (**A**) Representative images of hADSCs expressing GFP (green) demonstrated that hADSCs expressed circ_0011129. Scale bar: 100 μΜ. (**B**) Representative TEM images of 3D-circ-sEVs. Scale bar: 100 nm. (**C**,**D**) The size distribution and Zeta potential of 3D-circ-sEVs were measured by DLS (n = 3). (**E**) The design ideas of divergent and convergent primers. (**F**) qRT-PCR analysis of circ_0011129 and linear_0011129 mRNA after RNase R digestion in hADSCs (n = 3). (**G**) qRT-PCR analysis of circ_0011129 relative expression between hADSCs and circ_0011129-hADSCs (n = 3). (**H**) qRT-PCR analysis of circ_0011129 relative expression between 3D-sEVs and 3D-circ-sEVs (n = 3). Error bar, mean ± SD.

In order to improve the therapeutic efficacy of 3D-sEVs from hADSCs, we proposed to encapsulate our therapeutic nanovector system with circ_0011129, a miR-6732-5p adsorption sponge we previously reported to simultaneously inhibit the reduction of type I collagen and the denaturation and accumulation of elastin in HDF induced by UVA in both cell and animal photoaging models. To deliver circ_0011129 into the sEVs, we first generated the hADSCs overexpressing circ_0011129 and collected the sEVs from them cultured in a 3D bioreactor (3D-circ-sEVs). The picture showed that most of hADSCs successfully overexpressed circ_0011129 (Figure 3A,G). Next, we purified 3D-sEVs from circ_0011129-hADSCs and assessed the quality of these nanovesicles using transmission electron microscopy (TEM) and dynamic light scattering (DLS) analysis. Similar to 3D-sEVs, 3D-circ-sEVs had a spherical-like morphology with a membrane structure (Figure 3B), the zeta potential was around −15mV (Figure 3C), and the average diameter was around 100 nm (Figure 3D). Then, by designing divergent and convergent primers (Figure 3E), we verified that the target RNA expressed by hADSCs are circular structures, which was much more resistant to Rnase R digestion when compared to linear RNA controls (Figure 3F). Furthermore, we confirmed the existence of circ_0011129 from qPCR analysis of small RNA extracts, from 3D-circ-sEVs, confirming that circ_0011129 was loaded into 3D-circ-sEVs successfully (Figure 3H).

### 2.4. Preventive Effects of 3D-circ-sEVs on Photoaging In Vitro

**Figure 4:**
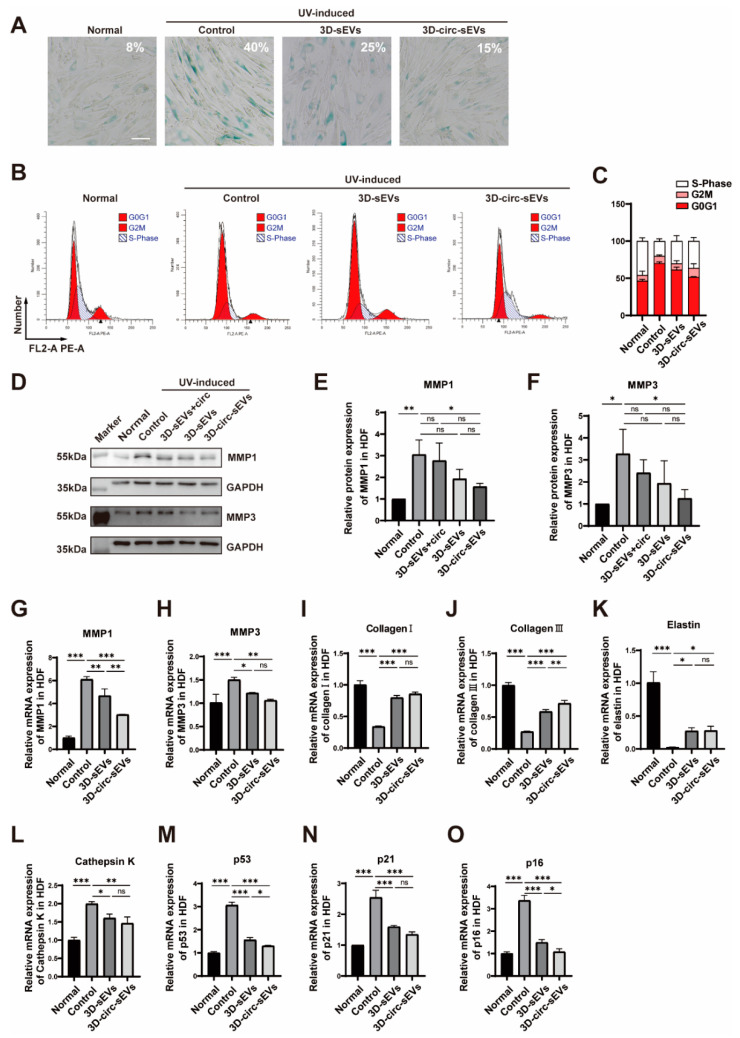
3D-circ-sEVs have a slight preventive effect on photoaging in vitro. (**A**) HDF cells were stained with a β-galactosidase staining kit. Blue color indicates senescent cells. Scale bars = 200 μΜ. (**B**) The cell cycle was analyzed in control group or treated group using flow cytometry on day 7. (**C**) Quantitative analysis of the effect of 3D-circ-sEVs on the cell cycle arrest alleviation (n = 3). (**D**) Representative blotting image of MMP1 and MMP3 in UVA-irradiated HDF cells after being treated by different sEVs. (**E,F**) Quantitative analysis of the effect of 3D-circ-sEVs on the protein expression of MMP1 and MMP3 (n = 3). (**G**–**O**) qRT-PCR analysis of the effect of 3D-circ-sEVs on the mRNA expression of MMP1, MMP3, Collagen Ι, Collagen ΙΙΙ, Elastin, Cathepsin K, p53, p21, and p16 in UVA-irradiated HDF cells (n = 3). One-way ANOVA and Tukey post hoc test analyses were performed. Error bar, mean ± SD. ns represents not significant. * *p* ≤ 0.05; ** *p* ≤ 0.01; *** *p* ≤ 0.001.

To investigate the role of 3D-circ-sEVs on photoaging in vitro, 3D-sEVs and 3D-circ-sEVs were both purified from hADSCs and then were co-incubated with HDF cells before starting UVA irradiation. A photoaging model was established by UVA chronic irradiation in HDF cells at a dose of 5 J/cm^2^/day for consecutive seven days and co-incubated with the sEVs at day 0 and 3. Cell senescence was detected by senescence-associated β-galactosidase (SA-β-gal) staining and flow cytometry analysis on day 7, respectively. The results showed that the percentage of SA-β-gal-positive cells was obviously decreasing when treated with 3D-circ-sEVs after 7 days of UVA irradiation (Figure 4A). Treatment of 3D-circ-sEVs reduced photoaging by decreasing SA-β-gal activity and UVA-induced cell cycle arrest in G1 phase in fibroblasts (Figure 4A–C). To further investigate photoaging-related gene expression, we conducted experiments with Western blot and qPCR (Figure 4E). MMP-1 and MMP-3 are involved in the regulation of collagen synthesis. The result showed that the expression of these protein was decreased in treatment groups comparing with UVA groups (Figure 4D–F) as well as the mRNA expression level (Figure 4G–H). qPCR analysis confirmed that mRNA expression of the proteins associated with cell morphology, including elastin and type Ι and ΙΙΙ collagen, reduced significantly after 7 days of consecutive UVA irradiation. As expected, the synthesis of collagen and elastin was enhanced for all treatment groups, especially for the 3D-circ-sEVs groups (Figure 4I–K). Our team previously discovered that cathepsin K is an enzyme involved in collagen degradation [28]. In this study, the mRNA expression of cathepsin K was up-regulated after UVA irradiation and relieved after treatment (Figure 4L). During cell cycle arrest, the p53 signaling pathway was activated. The mRNA expression of p53, p21, and p16 in UVA groups was significantly higher than control and treatment groups (Figure 4M–O). However, 3D-sEVs showed a limited repair and regulation capacity overall. 3D-circ-sEVs are well known for their ability to promote skin cell proliferation, and ameliorate skin photoaging in vitro. Taken together, treatment of 3D-circ-sEVs reduced photoaging by decreasing SA-β-gal activity and cell senescence or photoaging-related gene expression levels in fibroblasts.

### 2.5. Preventive Effects of 3D-circ-sEVs on Oxidative Stress Damage In Vitro

**Figure 5:**
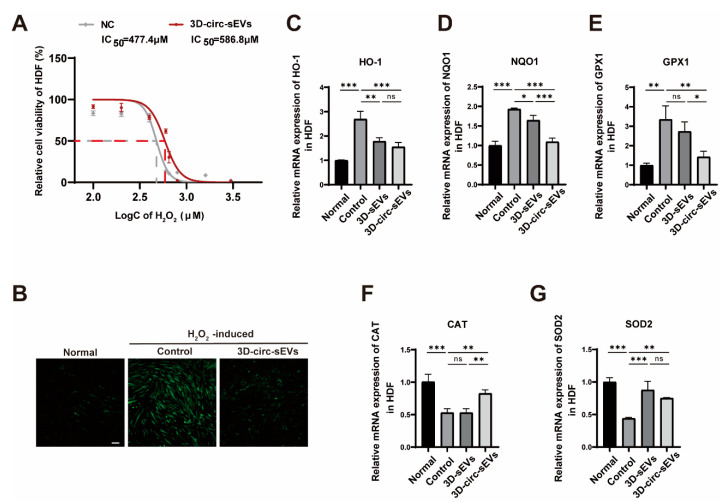
3D-circ-sEVs have a slight preventive effect on oxidative stress induced by H_2_O_2_ in vitro. (**A**) Relative cell viability of HDF treated with or without 3D-circ-sEVs tested by CCK-8. (**B**) Representative images showed the level of ROS in HDF cells treated with or without 3D-circ-sEVs. Scale bar: 100 μΜ. (**C**–**G**) qRT-PCR analysis of the effect of 3D-circ-sEVs on the mRNA expression of HO-1, NQO1, GPX1, CAT, and SOD2 in H_2_O_2_-induced oxidative stress HDF cells (n = 3). One-way ANOVA and Tukey post hoc test analyses were performed. ns represents not significant. Error bar, mean ± SD. * *p* ≤ 0.05; ** *p* ≤ 0.01; *** *p* ≤ 0.001.

Similar to photoaging, oxidative stress damage is also caused by the massive production of intracellular ROS. In this study, we used hydrogen peroxide (H_2_O_2_) to create an oxidative stress model in vitro to explore whether 3D-circ-sEVs could play a preventive role in the hydrogen peroxide oxidative stress damage. First, HDF cells were co-incubated with or without 3D-circ-sEVs for 24 h, and cultured for 6 h in H_2_O_2_ at a series of increasing concentrations. Then CCK-8 assay was applied to analyze HDF cells viability. The result showed that the IC_50_ of H_2_O_2_ to HDF cells increased from 477.4 μM to 586.83 μM (Figure 5A). 3D-circ-sEVs showed a slight preventive effect in the oxidative stress damage. Moreover, we detected ROS level of all groups after cultured for 12 h in H_2_O_2_ at 500 μM. The pictures showed that 3D-circ-sEVs can dramatically reduce the level of ROS induced by H_2_O_2_ (Figure 5B). To address whether 3D-circ-sEVs inhibit the activation of inflammatory pathways, we extracted the total cellular RNA to verify the mRNA expression of inflammation-related proteins upon different treatment. The results showed that 3D-circ-sEVs significantly inhibited the expression of HO-1, NQO1, and GPX1, while up-regulating the expression of CAT and SOD2 to reduce inflammation (Figure 5C–G). Therefore, 3D-circ-sEVs also have a preventive effect on cellular oxidative stress induced by H_2_O_2_.

## 3. Discussion

An important reason for the accelerated aging of photoaging skin is the degradation of collagen and the deposition of elastin. UVA irradiation induces the production of ROS, which can increase elastase activity and then degrade elastin, resulting in reduced skin thickness and elasticity, poor skin water retention, and wrinkle formation [2]. By detecting the ROS generated by HDF cells after UVA irradiation, the results showed that the hADSCs sEVs in 3D bioreactor culture had stronger anti-oxidative stress ability. It has been previously reported that sEVs secreted by HDF cells in 3D spheroids culture have strong resistance to photoaging, and the miRNAs in the exosomes play a key role. Therefore, we sequenced the miRNAs in 3D-sEVs and found that compared with traditional 2D culture, significantly changed miRNAs were enriched in cell activities related to apoptosis, cellular senescence, and inflammation under GO analysis. Cell cycle arrest is a major feature of cellular senescence, which involves activation and inhibition of the p53 signaling pathway. KEGG results showed that significantly changed miRNAs were enriched in p53, Ras, Wnt, and other pathways, indicating that 3D-sEVs have anti-aging functions. Similar to UVA-induced oxidative stress, H_2_O_2_ also induces cellular oxidative stress and is a more widely used model of oxidative stress [29]. To broaden the applicability of 3D-circ-sEVs, we assessed their protection against H_2_O_2_-induced oxidative stress. The results showed that 3D-circ-sEVs have a certain protective function on HDF cells, suggesting that they may have applications in other diseases involving H_2_O_2_ oxidative stress.

Our research group is the first to carry out the research on circRNA and skin photoaging. We recently found that there were 128 differentially expressed circRNA in HDFs chronic light injury model through circRNA chip technology. Moreover, it was verified that circ_0011129 has anti-photoaging effect in HDF cells [25]. However, the instability in vivo and low delivery efficiency to the target cell limited their widely application. sEVs have been identified as potential delivery vehicles of RNA reagents as they can protect the loaded RNAs from RNase and improve delivery efficiency to the target cells. The circular structure of circular RNA is the key to its stability [16]. To this end, after overexpressing circ_0011129 in hADSCs using lentiviral vectors, we designed divergent and convergent primers to demonstrate that the overexpressed RNA is circular rather than linear structure. Additionally, not all small molecules are encapsulated and transported out of cells during the production of sEVs [30,31], and there is currently no effective method to transform circRNAs to be encapsulated by sEVs according to the authors’ knowledge. We validated that circ_0011129 was indeed carried in 3D-circ-sEVs in our experimental approach. Moreover, sEVs can protect the circ_0011129 from hydrolysis by RNase R, likely resulting in the improved stability of the circ_0011129 in vivo. Consistently, we found that 3D-sEVs loaded with circ_0011129 showed a stronger protective effect against photoaging than the same amount of 3D-sEVs alone.

Previously, we found that circ_0011129 acts as a miR-6732-5p adsorption sponge to inhibit the reduction of type I collagen and the denaturation and accumulation of elastin in HDF exposed to UVA at the same time [25]. Consistent with previous studies, circ_0011129, delivered into HDF cells using 3D-sEVs, interfered with the process of cell photoaging by decreasing SA-β-gal activity, preventing the expression of MMP1, MMP3, and Cathepsin K, and accelerating the expression of collagen I, collagen III, and elastin. Overall, these results reveal that, as a natural membrane delivery system, hADSCs-derived sEVs in 3D bioreactor culture present themselves as an excellent alternative biological vector with advantages that can also dramatically enhance the therapeutic effects of loaded circRNA drugs.

**Figure 6:**
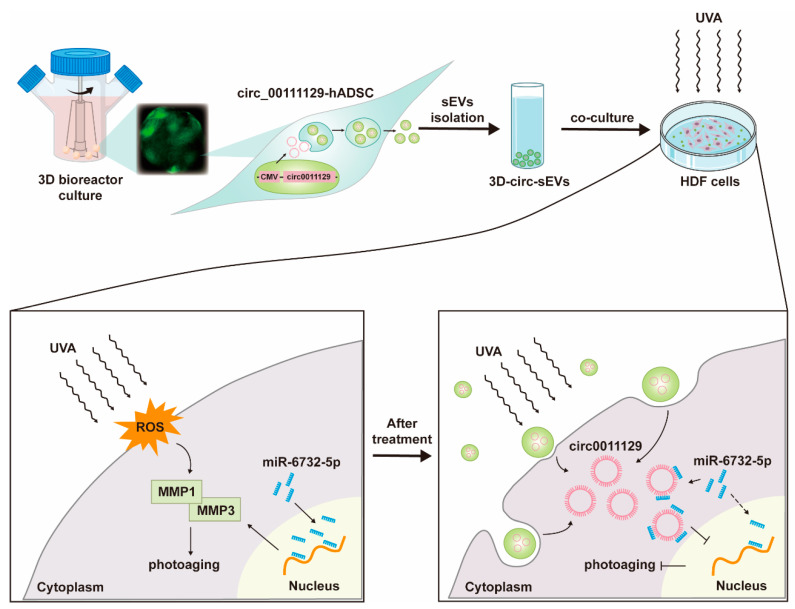
Schematic illustration of the mechanism of interference with the process of cell photoaging. 3D-circ-sEVs were purified from condition medium of circ_0011129-hADSCs in 3D bioreactor culture. Then, HDF cells were co-culture with 3D-circ-sEVs before or during successive UVA irradiation. Ultimately, circ_0011129 were accumulated in cytoplasm, adsorbing miR-6732-5p to inhibit its binding to target RNA. These effects contribute to interfere with the process of cell photoaging.

In conclusion, we designed a method to deliver circ_0011129 using hADSCs-derived sEVs in 3D bioreactor culture (Figure 6). The collected 3D-circ-sEVs can effectively and safely interfere with the process of cell photoaging and protect cells from UVA radiation damage. It provides a potentially promising therapeutic strategy for the protection and treatment of skin photoaging.

## 4. Materials and Methods

### 4.1. Isolation, Characterization, and Differentiation of ADSCs

Subcutaneous adipose tissue samples were harvested from the women who received liposuction, after informed consent was granted. Ethics approval was given from the medical ethics committee of The Third Affiliated Hospital of Sun Yat-sen University, China. Adipose tissue samples were Digest with 1% collagenase I and shaken in 37 °C for 40 min. After stopping digestion and removing connective tissue, the cell pellet was resuspended in Dulbecco’s Modified Eagle Medium F12 (DMEM-F/12, BI) with 20% fetal bovine serum (FBS, BI) and seeded in T25 culture flasks. The phenotype profile of hADSCs (passages 1 to 3) was evaluated through flow cytometry analysis by using cluster designation 34 (CD34), CD45, CD90, and CD105 (BioLegend, San Diego, CA, USA). The differentiation of hADSCs to adipocytes and osteocytes were tested, respectively, by using the adipogenesis induction differentiation kit (Cyagen Biosciences, Santa Clara, CA, USA) and osteogenesis induction differentiation kit (Cyagen Biosciences).

### 4.2. Cell Lines and Cell Culture

HEK-293T cells (human embryonic kidney cell lines) and HaCaT (human skin epidermal cell lines) were purchased from American Type Culture Collection (ATCC). HDF cells (human dermal fibroblasts cell lines) were a gift from professor Wenbin Deng. Cells were cultured in Dulbecco’s modified Eagle’s medium (DMEM, BI) supplemented with 10% FBS (ExCell Bio, Shanghai, China) and 1% penicillin/streptomycin (P/S, corning) at 37 °C. hADSCs were cultured in DMEM-F/12 supplement with 10% FBS (BI), 2% P/S and 10ng/mL basic fibroblast growth factor. For the 3D bioreactor culture of hADSCs, a 3D FloTrix miniSpin bioreactor (CytoNiche Biotech, Beijing, China) based on microcarriers was utilized according to the manufacturer’s instructions.

### 4.3. Plasmids and Stable Cell Lines

Plasmids of circ_0011129 (pLC5-ciR-puro-0011129-GFP) were purchased from Guangzhou Geneseed Biotech Co., Ltd. (Guangzhou, China). To obtain stable expression clones, hADSCs were transfected with lentivirus containing target gene, purchased from Hanbio Tech (Shanghai, China), and selected with puromycin (2 μg/mL).

### 4.4. Small Extracellular Vesicles Isolation and Characterization

sEVs were collected from cell culture supernatants by several density gradient centrifugation. When the cell density at 70–80%, the medium with 0.5% sEVs-free FBS was replaced and the cell culture supernatant was collected after culturing for 48 h. Then, it was centrifuged at 500× *g* for 10 min, 2000× *g* for 20 min, and 10,000× *g* for 40 min at 4 °C successively to remove cells, dead cells, and cells debris. Subsequently, the supernatant was centrifuged at 120,000× *g* for 70 min at 4 °C, followed by PBS washing and centrifuged at 120,000× *g* at 4 °C for another 70 min. Finally, hADSC-sEVs pellets were resuspended in pre-cooled PBS in a suitable volume and stored at −80 °C immediately for the following experiments. The morphology of sEVs was observed by a transmission electron microscope (TEM). The size distribution and Zata potential of sEVs were determined using a NanoBrook 90 Plus PALS (Brookhaven instruments, Holtsville, NY, USA).

### 4.5. UVA Irradiation of HDFs

Before the irradiation, HDF cells were incubated with 5% DMEM with or without sEVs at 50 ug/mL for 24 h. Then, cells were washed and added a thin layer with PBS before the exposure to UVA. For acute and chronic photoaging, the irradiation dose of UVA was 15 J/cm^2^ for one time or 5 J/cm^2^/day for 7 days, respectively.

### 4.6. Cell Viability Assay

Cell Counting Kit-8 (CCK-8) was applied to analyze HDF cells viability. HDF cells were seeded in 96-well plates and co-cultured with cell-conditioned media for 24 h. After 10 J/cm^2^ dose of UVA irradiation, CCK-8 reagent (Ape × Bio) was added and the absorbance was measured at 595 nm.

### 4.7. Detection of Cellular ROS by Fluorescence Microscopy 

After being treated with different CM, the medium of HDF cells was aspirated and washed once with PBS; then, an appropriate amount of fluorescent probe DCFH-DA (Sigma, St. Louis, MO, USA) working solution prepared by serum-free culture medium was added. HDF cells were placed in 37 °C for 20 min in dark, then washed twice times with PBS gently, and exposed to a 15 J/cm^2^ dose of UVA. Immediately observed under a fluorescence microscope at 488 nm. Fluorescence intensity was quantified using ImageJ software.

### 4.8. Western Blotting

Cells and sEVs were lysed with RIPA lysis buffer (Beyotime, Haimen, China) containing protease inhibitor (PI, Roche, Basel, Switzerland). The concentration of the protein was determined using a bicinchoninic acid (BCA) protein assay reagent (Beyotime). Equal amounts of protein were separated by 10% sodium dodecyl sulfate polyacrylamide gel electrophoresis (SDS-PAGE) and transferred to polyvinylidene fluoride (PVDF) membranes. After blocking, each small segment of the membrane was incubated with primary anti-bodies of MMP1 (1:1000, Proteintech, Singapore), MMP3 (1:1000, Affinity, West Bridgford, UK), and GAPDH (1:1000, Abmart, Berkeley Heights, NJ, USA) overnight at 4 °C followed with diluted corresponding secondary HRP-conjugated anti-bodies at room temperature for 1 h; then, detection was carried out using enhanced chemiluminescence reagent (ECL) (Protein Tech, Wuhan, China).

### 4.9. RNA Isolation and qPCR Analysis

Total RNA was extracted from cells using an EZ-press RNA purification kit (B0004D, EZBioscience, Roseville, MN, USA). miRNAs of 2D-sEVs and 3D-sEVs were isolated using the Exosome RNA Purification Kit (EZB-exo-RN1, EZBioscience). RNA concentration was determined by NANODROP ONE (Thermo Fisher Scientific, Waltham, MA, USA). The cDNA was synthesized by using EasyScript All-in-One First-Strand cDNA Synthesis SuperMix for qPCR (AE341-02, TransGen Biotech, Beijing, China) and then quantified by qPCR using PerfectStart Green qPCR SuperMix (TG-AQ601-02, TransGen Biotech) with LightCycler^®^ 96 (Roche). All processes were carried out by the manufacturer’s protocols. Relative gene expression folding changes were identified with the 2^−ΔΔCt^ method. All the primers used in this study are listed in Table 1.

### 4.10. Stability Determination of circ_0011129

To determine the stability of circ_0011129, the total RNAs of hADSCs were digested with 1U/ug RNase R (R0301, Guangzhou Geneseed Biotech Co., Ltd., Guangzhou, China) at 37 °C for 10 min and reversely transcribed into cDNA using TransScript One-Step gDNA Removal and cDNA Synthesis SuperMix (AT311-03, TransGen Biotech, Beijing, China) with random primers according to the manufacturer’s instructions.

### 4.11. β-Galactosidase Staining

HDF cells was stained using Senescence β-Galactosidase Staining Kit (Beyotime) according to the protocol and incubated overnight in incubator without CO^2^ at 37 °C. The staining results were observed under an inverted microscope.

### 4.12. Statistical Analysis

All experiments were performed independently at least three times. All data were expressed as the mean ± SD. Data analysis and processing were performed using GraphPad Prism Ver 8.0.2 software. The statistical significance was determined by a one-way analysis of variance (ANOVA) followed by Tukey’s test, and statistical significance was indicated (* *p* ≤ 0.05; ** *p* ≤ 0.01; *** *p* ≤ 0.001).

## Figures and Tables

**Table 1:** The sequences of the qPCR primers.

Gene	Forward Primer Sequence 5′→3′	Reverse Primer Sequence 5′→3′
H-*GAPDH*	AGAAGGCTGGGGCTCATTTG	GCAGGAGGCATTGCTGATGAT
H-*MMP1*	AAAATTACACGCCAGATTTGCC	GGTGTGACATTACTCCAGAGTTG
H-*MMP3*	AGTCTTCCAATCCTACTGTTGCT	TCCCCGTCACCTCCAATCC
H-*COL1A1*	GAGGGCCAAGACGAAGACATC	CAGATCACGTCATCGCACAAC
H-*COL3A1*	GGAGCTGGCTACTTCTCGC	GGGAACATCCTCCTTCAACAG
H-*Eastin*	GGAGCTGGCTACTTCTCGC	GGGAACATCCTCCTTCAACAG
H-*Cathepsin K*	ACACCCACTGGGAGCTATG	GACAGGGGTACTTTGAGTCCA
H-*p53*	CAGCACATGACGGAGGTTGT	TCATCCAAATACTCCACACGC
H-*p21*	TGTCCGTCAGAACCCATGC	AAAGTCGAAGTTCCATCGCTC
H-*p16*	GATCCAGGTGGGTAGAAGGTC	GATCCAGGTGGGTAGAAGGTC
H-*HO-1*	AAGACTGCGTTCCTGCTCAAC	AAAGCCCTACAGCAACTGTCG
H-*NQO1*	GAAGAGCACTGATCGTACTGGC	GGATACTGAAAGTTCGCAGGG
H-*GPX1*	CAGTCGGTGTATGCCTTCTCG	GAGGGACGCCACATTCTCG
H-*CAT*	TGGAGCTGGTAACCCAGTAGG	CCTTTGCCTTGGAGTATTTGGTA
H-*SOD2*	GCTCCGGTTTTGGGGTATCTG	GCGTTGATGTGAGGTTCCAG
H-*linear_0011129*	TTCCGGGCCCAGGTC	CATCACCACTGCCAGGTT
H-*circ_0011129*	GAGGACTTCACTCGGAGAGG	CACCACTGCCAGGTTGTCT

## Data Availability

The data presented in this study are available on request from the corresponding author.
